# Methodological considerations when assessing the Burden of Disease due to injuries: Robby De Pauw

**DOI:** 10.1093/eurpub/ckac129.633

**Published:** 2022-10-25

**Authors:** R De Pauw

**Affiliations:** Sciensano, Brussels, Belgium

## Abstract

Measuring the burden of disease due to injury is a similar process compared to the disease burden. However, burden calculations for injuries impose an extra complexity for the non-fatal burden, as it is important to consider both the ‘cause’ and the ‘nature’ of the injury. Cause of injury (e.g., road injuries, falls, drowning) have historically been used for assigning cause of death as opposed to the ‘nature’ of injury, which more directly specifies the pathology that resulted in death or disability. However, precisely estimating the disability as a result of an injury requires a mapping of, for example, individuals that have suffered a fracture hip (‘nature’ of the injury) because of falling (‘cause’ of the injury). This process would require a matrix of the nature-cause relation of injuries, which are only scarcely available, because they required dual-coded data registers. The presentation will zoom into the differences between disease and injury estimations and will appraise the methodological considerations for the estimations of injury-related burden of disease estimates.

References

James, S. L., Castle, C. D., Dingels, Z. V., Fox, J. T., Hamilton, E. B., Liu, Z., ... & Fomenkov, A. A. (2020). Estimating global injuries morbidity and mortality: methods and data used in the Global Burden of Disease 2017 study. Injury Prevention, 26(Suppl 2), i125-i153.

Haagsma, J. A., James, S. L., Castle, C. D., Dingels, Z. V., Fox, J. T., Hamilton, E. B., ... & Mahotra, N. B. (2020). Burden of injury along the development spectrum: associations between the Socio-demographic Index and disability-adjusted life year estimates from the Global Burden of Disease Study 2017. Injury prevention, 26(Suppl 2), i12-i26.

